# Pain, instability, and familial discord: a qualitative study into women who use drugs in Malaysia

**DOI:** 10.1186/s12954-015-0086-6

**Published:** 2015-11-05

**Authors:** Fifa Rahman, Priya Lall, Sarah Iqbal, B. Vicknasingam

**Affiliations:** Malaysian AIDS Council, No. 12, Jalan 13/48A, Sentul Raya Boulevard, 51000 Kuala Lumpur, Malaysia; University of Malaya, Medical Faculty, 50603 Kuala Lumpur, Malaysia; Centre for Drug Research, Universiti Sains Malaysia, 11800 Penang, Malaysia

**Keywords:** Women who use drugs, Malaysia, Instability, Family, Childhood, Marriage, Support services

## Abstract

**Background:**

Out of 20,887 persons who use drugs that came into contact with the National Anti-Drugs Agency (NADA) officials in the year 2013, 3.2 % were women. Because women who use drugs (WWUD) are often a hidden population, this may be an underestimate. International literature shows that women who use drugs face increased risk of HIV, intimate partner violence, and mental health issues. Similar literature in Malaysia is lacking, and thus, the objective of our study was exploratory in nature.

**Methods:**

Thirty-eight women who use drugs were interviewed using a semi-structured topic guide in Kelantan, Penang, Johor, Kuala Lumpur, and Selangor. Locations were chosen purposively. Nineteen women were interviewed individually and the remaining 19 were in focus group discussions (FGDs). All interviews were transcribed verbatim, translated to English, and analyzed with NVivo.

**Results:**

Median age of respondents was 35.5 years old, 89.5 % ethnic Malays, majority having married below the age of 20, and were of low socioeconomic backgrounds. Youngest age of initiation into drug use was 9 years old. Most reported is inhalation of amphetamine-type substances. Seven reported ever injecting. Three themes emerged: (a) repeating patterns of fluid family structures and instability; (b) “pain” and “difficulty” as features of home life; and (c) seeking marriage as a source of stabilization and practices of power within those marriages. Respondents often came from very fluid family environments and married to find stability, only to be drawn into a similar cycle. None of the women who had been separated from their children either institutionally, by family members, or by third parties, had accessed legal recourse for the loss of their parental rights.

**Conclusion:**

Unstable familial relationships or environments contributed to earlier initiation of drug use which raised questions about support services for WWUD and children who use drugs. Respondents were drawn into unstable and/or abusive relationships, perpetuating social inequalities that marked their own familial environments during childhood. These findings support the need for additional services to support the unique needs of WWUD, including domestic violence services, financial and life skills, parental rights assistance, and empowerment programs.

## Background

Only 143 women who use drugs (WWUD) were detected in Malaysia in 2013 compared to 7721 males [1]. The hidden nature of WWUD and the inadequate interventions for them make it difficult to access this population [2]. WWUD face more stigma than men who use drugs because their drug use is seen as contravening the natural roles of women in society, i.e., as “mothers, the anchors of their families, and caretakers” [3, 4]. Women experience addiction and its contributing factors differently than men, and that they have unique needs; for example, those related to sexual and physical violence and pregnancy care [5, 6].

There is a lack of literature documenting the experiences of WWUD in Malaysia. A 2014 study of 120 women held in compulsory drug detention in northern Malaysia showed that 39.5 % of respondents initiated drug use from the ages of 15–20, but did not examine contextual and socioeconomic factors of drug use [7]. In 2014, 20 % of new HIV infections in Malaysia were women [8].

Numerous studies predominantly conducted in developed countries found the following. Women who use drugs are at a higher risk of contracting HIV due to biological, behavioral, and structural reasons [9]. Women who inject drugs are often injected by their male partners and so are often “second on the needle”, which increases risk of HIV transmission [10]. WWUD are at greater risk of psychological disorders [11]. A study conducted among 118 WWUD in Barcelona, Spain, estimated that participants with depressive disorders face a 2.42 times greater risk of intimate partner violence compared with respondents without depressive disorders [12]. Family physicians treating WWUD report having to deal with evident experiences of trauma and violence [13]. Among WWUD respondents in a study conducted in New York, childcare was cited as a barrier to enrolment in drug dependence treatment [14].

Studies show that WWUD have different behavioral patterns than men who use drugs. A study done among 672 females who inject drugs in 10 developing countries showed that females were more likely to engage in risky practices in the context of a sexual relationship with a primary partner [15]. There are relatively fewer studies conducted in Southeast Asia on WWUD, especially in Muslim contexts. A study conducted among WWUD in Java, Indonesia, which is culturally and religiously congruent to Malaysia, found that initiated to drug use could occur as a demonstration of loyalty to a male partner who used drugs [4].

Given that the majority of respondents were Malay-Muslim, cultural norms may be relevant for consideration [16]. A 1997 study on social support and mental health among rural and Malay women detailed the influence of Islam on Malay families as part of the Malay culture and identity, and stated that within these families, gender roles are often patriarchal and fundamentalist [16]. Later in the same article, the author noted that rural respondents felt that their environments were not conducive for positive mental health conditions. Mamat (1991) in her book on the role and status of Malay women in Malaysia described that women are regarded as running the household but that the husband holds the position of prime authority in the household [17]. In Indonesia, a culture with many similarities to Malay-Muslims, Jacubowski (2008) in examining heterosexual marriage, not only discussed the emphasis on the “natural” roles of women as wives and mothers in Indonesian culture, but also the fact that many participants considered marriage as an obligatory act [18].

WWUD in Malaysia are subject to drug laws that are some of the most punitive in the world, including incarceration and judicial corporal punishment for drug use and possession, and the death penalty for drug trafficking [19]. State-endorsed harm reduction services, however, operate tangentially to these laws. In fact, from 2012 to 2014, Malaysia had the steepest increase in scale-up of needle-and-syringe exchange programs (NSEPs) in Asia [20]. While voluntary drug treatment services exist in the form of Cure and Care Service Centres (CCSCs), abstinence-based compulsory detention centers called Cure and Care Rehabilitation Centres (CCRCs) continue to operate. There is one CCRC nationwide that is women-only, but there is no documented evidence in regard to services specifically to cater to unique needs as aforementioned, including sexual and physical abuse and pregnancy care. Young people who use drugs in Malaysia also risk being detained in “moral rehabilitation centres”. These centers do not incorporate adequate healthcare and social services, and have been deemed to be ineffective [21].

There is a dearth of research on the WWUD in general in Southeast Asia, therefore little is known on behavioral and structural factors driving drug use in this population. This study attempts to fill this gap through employing exploratory qualitative methods elicit responses regarding backgrounds, experiences with drug use, and experiences with support services, whether government-, private-, or NGO-provided. We sought to conduct in-depth interviews exploring these relationships, among others. We also sought to examine WWUD experiences with existing harm reduction services and whether those harm reduction services were addressing the respondents’ unique needs.

## Methods

The use of qualitative methods in this paper was guided by grounded theory; an inductive research strategy was used to develop the sampling framework and topic guide. In-depth interviews and focus group discussions were conducted by all authors in mostly urban settings in the states of Penang, Kelantan, Kuala Lumpur, Selangor, and Johor, to obtain qualitative data on backgrounds, family life, experiences of drug use, and experiences with support services. Locations were chosen purposively based on data on levels of drug use, and proximity to harm reduction services, so as to enable outreach workers to recruit respondents. Respondents were not necessarily existing clients of these services. States were systematically selected in the North (Penang and Kelantan), Central (Kuala Lumpur and Selangor), and South (Johor) of Peninsular Malaysia so as to ensure access to a wide range of WWUD across the country. Participants were 38 adult females, 19 of which were interviewed individually using a semi-structured topic guide, and the remaining 19 were in focus groups (nine respondents in FGD 1, five respondents in FGD 2, and five respondents in FGD 3). We sampled respondents until saturation (Table 1).

**Table 1:** Sites and data collection methods (*N* = 38)

Site	Data collection method
Penang	In-depth interviews (*N* = 5)
	Focus group discussion (*N* = 9)
Klang Valley (Kuala Lumpur and Selangor)	In-depth interviews (*N* = 4)
	Focus group discussion (*N* = 5)
Kelantan	In-depth interviews (*N* = 4)
	Focus group discussion (*N* = 5)
Johor	In-depth interviews (*N* = 6)

Topic guides explored the following domains: (a) childhood experiences, (b) drug use history and causes of drug use, (c) current and past family life, and (d) experiences with drug treatment/harm reduction services. These domains were chosen due to the lack of published research on the backgrounds and experiences of WWUD in Malaysia, and were chosen based on consultations with stakeholders including WWUD. During the course of data collection, topic guides were refined according to themes emerging from interviews.

All participants were recruited through outreach efforts except for interviews conducted in Penang. Outreach workers under the Malaysian AIDS Council contacted potential study participants, explained the goals of the study, and obtained verbal consent from those who were interested. Interviews conducted in Penang were done with the assistance of the National Anti-Drugs Agency. The National Anti-Drugs Agency is a government agency responsible for the prevention of drug use. Participants were informed that researchers from Malaysian AIDS Council and Universiti Sains Malaysia were conducting a study to learn about the lives of WWUD, including their childhoods and current family lives, and experiences with drug use. No identifying information was collected. All interviews were audio recorded. The study was approved by the Human Ethics and Research Board of Universiti Sains Malaysia.

Interviews in Kelantan were conducted with the assistance of persons proficient in the local dialect. The FGD in Selangor was conducted with the assistance of an international researcher. To facilitate understanding of interview content by the English-speaking researcher, all questions, answers, and discussions were translated by the local researchers between English and Bahasa during each interview. All individual interviews were conducted at harm reduction facilities except for one in a village house in Kelantan. One FGD was conducted at a drug treatment training facility, one FGD was conducted at a harm reduction facility, and the other FGD was conducted in a participant’s house. There was one group facilitator and one note taker for all interviews, and a translator present for the FGD conducted with the international researcher. The facilitator provided an introduction about the purpose of the group discussion, explained participant rights, anonymity and confidentiality of collected information, and established ground rules before beginning the discussion. Participants were given the opportunity to ask questions and express any concerns before each interview started.

Interview notes were initially collated and organized. Glaser et al.’s (1967) grounded theory was used to identify and code themes that emerged from the data [22]. Observations emanating from these notes were used to develop “open codes”, which were in turn used to categorize large sections of the data by authors FR and SI. The audio data was then transcribed and translated into English; it was not possible to back translate due to resource constraints. The process of translation was an additional component of analysis in which researchers with fluency in both Malay and English generated a set of codes related to social norms unique to Malay language. Transcripts were then analyzed through NVivo. All authors involved in this paper further refined codes to be reflective of core categories and link concepts. Areas of the text were also selectively coded to characterize relationships between categories. Finally, themes were identified through a carefully kept audit trail and comparison between core categories.

## Results

### Participant characteristics

The sample consisted of 38 women, ranging from 18 to 56 years old, with a median age of 31 years old. Racial-ethnic composition was 89.5 % ethnic Malay, 5.3 % Malaysian Indian, 2.6 % Malaysian Chinese, and 2.6 % Malaysian of Cambodian descent. Thirty-seven percent of respondents had completed secondary school education. Eighteen percent had never married. Among those who had married, majority were married below the age of 20. Six respondents reported undergoing intimate partner violence, and six respondents reported undergoing childhood violence.

The median age of first illicit drug use was 18 years old, with the lowest age of initiation of 9 years old. Many participants reported administering crystal methamphetamine through inhalation. There may have been slight regional variations in drug use as respondents in Kelantan mainly inhaled heroin and amphetamine-type substances. Seven women reported ever injecting drugs. Most respondents were recruited via Malaysian AIDS Council needle-and-syringe exchange program outreach workers (Table 2).

**Table 2:** Background characteristics of respondents (*N* = 38)

Characteristic	Percentage (%)
Age (median age = 31 years old)	
Younger than 30	44.8
30–40	31.5
40–50	18.4
Older than 50	5.3
Age of first illicit drug use	
Younger than 18	31.5
18–25	34.2
25–30	15.7
Older than 30	2.6
Unknown	16.0
Educational status	
Never schooled	2.7
Completed primary school	55.2
Completed secondary school	36.8
Completed tertiary education	0
Unknown	5.3
Marital status	
Never married	18.4
Married	39.4
Separated	0
Divorced	23.6
Widowed	0
Unknown	18.6
Has a child/children	50
Place of residence	
Urban	71
Rural	10
Unknown	19

### Themes from analysis

Three main themes emerged in the interviews in regard to WUD backgrounds: (a) repeating patterns of fluid family structures and instability, (b) acknowledged day-to-day pain and difficulty and its effect on quality of life, and (c) seeking marriage as a source of stabilization and practices of power within those marriages. We present and discuss these with illustrative quotes below.

### Theme 1: repeating cycles of fluid family structures and instability

“Chaotic” childhoods seemed to feature strongly in respondents’ accounts of factors leading to drug use [23]. From our analysis of the literature, it emerged that respondents experienced fluid family structures marked with divorce, remarriage (therefore, introduction of stepparents), and an assumption of the parental and guardianship role by extended family. Fluid family structures were often enabled by problematic drug use, and this also created an environment for intergenerational transmission of drug use:

*When I was a child my parents were poor/not well off (difficult), my stepdad beat me, smoked pot and beat me, beat me, beat me non-stop. When I was 10, I ran away. I was separate, my big sister was separate, my little sister was separate. 10 years old, I became bad, started taking drugs.*

*(Penang, 47 years old)*

It was also common that fluid and unstable family structures were accompanied by other structural obstacles. Poverty, violence, ill health, and migration permeated the respondents’ experiences. For a 24-year-old mother of two children in Johor, dropping out of school at the age of 14 out of necessity for survival was precipitated by parental abandonment and neglect. As an adult with a low level of education and few job skills, the respondent participates in survival crime.

*I dropped out of school. I looked for work, for food, everything on my own. I had to pay for things myself. My mum married someone else, you know. My dad died because of HIV, so I had to look for my own money because she didn’t pay for anything for us. Her husband didn’t give us anything either… When I was 18 I was already married, and my husband was just like me (using drugs). I didn’t know he was like that as well, and his parents didn’t like it at all because I’d gotten pregnant before marriage, and he wasn’t working so we made money selling other people’s coconuts, stealing what we can to survive.*

*(Johor, 24 years old)*

Changing family structures often resulted in frequent loss of familial support networks. A 30-year-old respondent from Kelantan related how she had been in three different family structures before the age of 18, and she expressed a preference for one. Respondents experienced constant displacement, and many felt that they were appendages, guests, and inconveniences to their new families.

*When I was small, I lived with my mum and my stepdad. When I was fourteen, I was sent to live with my grandmother. So I lived with my mum and my stepdad until eleven years old. After that I lived with my dad and my stepmum until I was fourteen. I wasn’t happy. I was only happy when I was with my dad.*

*(Kelantan, 30 years old)*

The above respondent (Kelantan, 30 years old) later underwent three divorces at ages 16, 17, and 26, and underwent traumatic separations from her children. Feelings of resignation and abandonment resonated from narratives of fluid family structures. This respondent yearned for her mother, and professed that she had given up with school due to having “too many problems”:

*My mum married someone else, she left me with my grandma. So at that time I wanted to go with her but I was forced to live with my grandma… I was just at a dead-end with schooling. I had too many problems. I was still small and I already got left behind, abandoned. My little sister was also still small. So I started selling vegetables with my older sister.*

*(Kelantan, 38 years old)*

The cycle of poverty and dysfunctional family structures continue and it seems that if no interventions are provided the same cycle with the children will repeat.

### Theme 2: acknowledged day-to-day pain and difficulty

The Malay words *sakit* (pain) and *susah* (difficult) repeatedly arose in interviews. In the Malay language, the word *susah* is often used to illustrate economic difficulty [24], in addition to the standard English definition of “not easy” or “hard to do” [25]. Conscious of the economic dimension/connotation of the word *susah* in the Malay, we found that experiences of pain and hardship were often linked to socioeconomic conditions.

*Like in those days of course my life was really difficult (susah). My mum worked sewing clothes. My dad drove a lorry. Because my dad had a gambling habit, the money from salaries was just never there. If he got his salary he wouldn’t come home. Two or three days would pass and he would come back and say that he didn’t get his salary. So only mum’s earnings were available for us kids. This went on until their divorce, where my dad said to my mum, ‘If you claim any monies/alimony from me I’ll take your children.’ So my mum never asked for a single cent from him.*

*(Johor Bahru, 33 years old)*

*My life was so very difficult. (susah) I didn’t work, I lived with my late grandmother. Because my family life was up and down and all over the place, like flotsam and jetsam, my parents were problematic, I had no interest in school, I didn’t know where my parents were, separated, that’s why I feel like it was extremely difficult.*

*(Johor Bahru, 40 years old)*

*We are poor people (susah). Fishermen can make money, but if there are no fish, then there’s no money. The problem is if I ask for money from him (husband), he starts complaining (making noise). So I’m lazy to bring it up.*

*(Kuala Lumpur, 55 years old)*

Experiences of pain were both physical and emotional, and parents were often the key actors or instigators of pain.

*My dad forced me to get married. (I got married at) sixteen. If I didn’t stop (taking drugs), my dad was the type that liked to abuse people… I would suffer (sakit) later on. So I had to listen to him. I got divorced, then he died.*

*(Kelantan, 36 years old)*

*Last time, I was like everybody else. I got involved in drugs because of stress. My dad started acting up. Like he took up with my niece, my cousin, went overboard and acted like man and wife with her, had sex. My cousin, I see her as my little sister. My dad had always advised me, don’t do this, don’t do that. He was my hero. But then he messed up. So I was under a lot of pressure. I don’t really know how far the effects on me are.*

*(Kelantan, 36 years old)*

Several respondents had been separated from their children; either by state authorities, by extended family members, or by third parties. These separations were a clear source of trauma and pain:

*Yeah. I saw him recently, they said they want to move him. I told them if you move him I can’t see him anymore, it’s too far… He’s my flesh and blood. My own child has gone far away, and I can’t see him. (Penang, 47 years old)*

*If I tell you about it now I’ll feel like crying. Because he’s my child, I carried him in my womb, I gave birth to him. But it’s okay.*

*(Selangor, Focus Group Discussion)*

Many participants acknowledged that unstable family structures often acted as gateways to drug use. Meanwhile, pain and difficulty resultant from environmental factors associated with their socio-economic condition and unstable family structured seemed to encourage continual drug use. A few participants believe that they used drugs to mitigate the impact of these environmental factors.

*It hurt so much thinking about how my mum never cared for me (‘ignored me’). I could go wherever I wanted and they didn’t care. Because I saw them doing that to me, I felt like doing drugs again. So I started doing drugs again and until I got married it was the same.*

*(Johor, 24 years old)*

### Theme 3: seeking marriage as a source of stabilization and practices of power within those marriages

Marriage was often perceived as a solution to unstable and perceived difficult childhoods or a way to establish a stable environment. While some respondents explicitly mentioned the role of parents in arranging these marriages, the majority was silent as to this fact. The reasons for early marriage were often related to avoidance or escape from particular circumstances or persons from their unstable childhoods. Interestingly, while this motive was prevalent, respondents often ended up in marriages with partners who used drugs. Some respondents felt that the marriages were rushed and ill-planned, and that but for their naïveté, they would not have married men who use drugs:

*My mum married someone else, she left me with my grandma. So at that time I wanted to go with her but I was forced to live with my grandma… I got married when I was 19. I wanted to follow in my mum’s footsteps, right? I couldn’t stand my stepfather, so I got married… I learned how to take heroin from him, who else? I was stupid! I was 19, you know!? Stupid. I didn’t even know what drugs were.*

*(Kelantan, 38 years old)*

*I didn’t know he took drugs too. I married him not because of love, but I was forced.*

*(Selangor, 31 years old)*

Circumstances that precipitated early marriage included a lack of productive or economic activity. A triple-divorced respondent who entered her first marriage at the age of 16 elaborated:

*No, because I wasn’t schooling, or anything, right? I sat at home with my grandma. So you know what parents are like, that’s why they told me to get married.*

*(Kelantan, 30 years old)*

Marriage was often discussed following discussions of economic circumstances, and although for some, it was not explicitly mentioned that one resulted in the other, for most respondents’ marriage was seen as a natural step in life. Early marriages were marked with instability, and often resulted in dissolution:

*I worked at someone’s house, domestic work. We’re poor people, miss, my late dad was a fisherman, if he made money then we ate. I helped the family a little bit. We were a poor family. Working as a domestic helper is cheap, 30 ringgit per month. So I helped a little bit. I did the laundry and washed people’s clothes. Sooner or later at 16 years old I got married. Early marriage. And then I got divorced. I got divorced at 16 as well. I was only married for 3 months, it wasn’t long.*

*(Kuala Lumpur, 55 years old)*

*I got married at 19. Of course I don’t have children, we got divorced after two months, how the hell was I supposed to have children?*

*(Penang, 21 years old)*

*I got married when I was 19… He doesn’t see our child, I don’t know where he gone, he’s just missing. I’ve never heard news, havent met, I don’t know any stories, I don’t know.*

*(Johor, 27 years old)*

Respondents’ narratives pertaining to marriage dissolution reflected power: for some, this emerged at the apex of marital conflict; for others, this emerged later. For one respondent interviewed in Kuala Lumpur but who grew up and married in the northern state of Terengganu, despite social conventions at the time being more accepting of polygamous marriages, discovering that she was a second wife post-marriage precipitated separation:

*When I married him, I was the younger (wife). Number 2. So in those days when we got married, the first wife didn’t have to sign. After we got married then I found out he had a wife already and I straight away fought with him. At first I didn’t know he had a wife. Until now he hasn’t divorced me. He left it just like that.*

*(Kuala Lumpur, 55 years old)*

We learned that despite instability transmitting to and penetrating each generation, decisions were made to avert similar instability away from offspring. For a 30-year-old three-time divorcee in Kelantan, the decision not to fight to get her children back from her husband was closely linked to her experiences with her stepfather as a child. She related through her tears:

*(My stepfather) treated me differently, and his own kids he treated differently. It was a given that I would always get beatings. If I don’t do something properly then I’ll get it… I’m thinking this way because I’ve lived with a stepfather before. The wife has to follow the husband, right? I don’t want my kids to go through the same thing.*

*(30 years old, Kelantan)*

Inherent in this was a sense of power, but intertwined with sacrifice. Other studies have shown that WWUD want to “break the cycle” [23], and in our study, the exercise of power in dissolution of marriages was tinged with great personal sacrifice:

*The second one is from a different father. I got married when I was 16… I worked part time with Benson & Hedges. Cigarettes. After that I was not allowed to work, my husband didn’t allow me to work, I rested & became a housewife… Then we got divorced. It just wasn’t fated. There wasn’t any problems. My ex husband didn’t use drugs. I made the decision, asked him to let me go because I didn’t want to trouble anybody. I took things into my own hands.*

*(Johor Bahru, 40 years old)*

One respondent from the state of Johor in the south described how she had divorced her first husband by way of the Islamic law concept of *fasakh*, i.e., where a woman asks for a divorce on grounds of the husband’s inability to provide (sexually, financially, or emotionally), disability, disappearance for a long time, apostasy, abuse, or the husband does not fulfill religious obligations [26]. The process is more cumbersome than when the husband requests the divorce (*talaq*), where there is less or no emphasis on reason for divorce. The respondent was also married in Thailand, and had to travel to Thailand to obtain the *fasakh* decree.

## Discussion

This qualitative study explored themes of poverty, pain and trauma, early marriage, and instability and contributes to the knowledge surrounding contributing factors to drug use among Southeast Asian and Muslim WWUD and to a certain extent young females who use drugs, with the objective of elucidating gaps in services in Malaysia. Given the lack of research on backgrounds and in-depth of WWUD in Southeast Asian countries and specifically among Muslim women, we aimed to inductively draw out themes pertaining to these women’s lives.

Firstly, many participants spoke at length about how familial factors first acted as a gateway to drug use and then later enabled continued drug use. This could be in part due to the fact that these participants took a holistic view of their drug use, seeing it not just resulting from their individual behavior but also from environmental factors. This is consistent with studies elsewhere [27]. Uniquely, our study predominantly features drug use within a familial context as opposed to injection in public environments.

Secondly, instability due to fluid family structures resulted in a loss of familial support networks. The loss of these networks led to evident pain, difficulty, and neglect during their childhoods. Poverty exacerbated many respondents’ lives, meaning that many had to drop out of school and begin work for survival. We found striking similarities between our results and results in a study conducted in Vancouver’s Downtown Eastside among 27 postpartum women accessing harm reduction services. This study by Torchalla et al. observed that “normality and daily routines were missing in the lives of most participants, and many of them were unable to complete school, get an education, and take up employment. Their childhood was often chaotic and characterized by abuse and neglect and it continued to be so in adulthood.” [23] This trauma was observed to be transgenerational.

Also observed to be transgenerational in our study was marital instability. There were evident experiences of pain associated with parental marital instability, and as a result of parental marital instability, there was a lack of parental oversight, which allowed respondents to go out and take drugs. Studies have oft-remarked that parental divorce is a risk factor for marital dissolution [28]. Among our respondents, we suggest that transgenerational marital instability was a result of a number of factors, including parental marital instability, economic adversity, and fluid family structures. In addition to this, studies have shown children who undergo multiple transitions in family structures may fare worse that children raised in stable two-parent, and even stable single-parent families [29]. Some studies have shown that a child’s transition from a two-parent family to a single-parent family is associated with lower school engagement, poorer cognitive achievement, and more behavior and emotional problems. [30] Other studies show that children in single-parent families use significantly more inhalants, marijuana, and amphetamines than peers from intact dual-parent families [30].

Economic adversity was seen to compound and exacerbate this instability. Studies among low-income mothers show that there is an increased likelihood of neglect [31]. A combination of these factors seems to create an environment conductive to problematic drug use. It is pertinent that these women are reachable by needle-and-syringe outreach workers and drug treatment services but not welfare services suggest that real gaps exist. These gaps could include insufficient capacity of harm reduction outreach workers to refer to alternative services, the lack of gender-sensitive services incorporated within harm reduction structures, or a lack of outreach work by state welfare services, among others.

A majority of respondents were married below the age of 20. Many women suggested that their youth/naïveté was the reason for early marriages, and that but for their naïveté, they would not have entered into partnerships with drug-taking partners. As a majority of respondents were Malay-Muslim, it may be useful to consider the cultural context of expectations among Muslims to marry and raise families. Jones et al. (2011) comment that while marriage ages have increased in Malaysia and Indonesia, in both these countries, the cultural imperative remains. For example, the authors detail the “moral panic” that arises every time statistics are released showing increasing numbers of Malay-Muslim women remaining single into their 30s. The authors comment: “The underlying assumption is that to remain single is a disaster for a woman… it is a duty for Muslims to marry and raise a family” [32].

Marriage is viewed as a natural step for many Malay women, but norms about early marriage have transformed. In a study conducted in Indonesia, a country with similar cultural background and norms, among WWUD, early marriage was seen to play an important role in terms of increasing the vulnerability of women to HIV, not only due to structural factors, but also due to biological factors and factors related to gendered power relations and gender inequality [18]. Marriage was perceived by the respondents in our study to be a solution to instability, but in most cases this hope did not materialize. These findings are potentially important for the discourse on early marriage on Malaysia, given cases of early marriage among Malay-Muslim girls are rising [33]. Also in Indonesia, a study among 5816 ever married women showed that level of education was the strongest predictor of early marriage [34]. Given that there are associations in our study and others between early marriage, risk of HIV infection, and drug-using behavior, more research is needed to determine strength of associations between early marriage, marital instability, and drug use.

Crucial to the topic of harm reduction is that given these women are most accessible by needle-and-syringe outreach workers, it may benefit state authorities to incorporate gender-sensitive welfare services into harm reduction services, including facilitation to return to schooling, financial skills workshops for women, domestic violence education and counseling, and a number of different interventions to enable girls and young women to make more informed choices regarding early marriage.

These women and girls are arguably in a “weak” position, not due to the physical vulnerabilities, but due to structural vulnerabilities rooted in poverty, childhood adversities, and early marriage. The idea that early marriage is a solution to instability often renders the women dependent on a male partner, which attracts a discussion on gendered power relations and decision-making. This is compounded by beliefs prevalent in many cultures that grant men more power to make decisions, earn higher incomes, act against their partners’ wishes, and control their partners’ actions, hence resulting in a greater likelihood of women having less power than male partners in intimate relationships [35]. These have implications for HIV risk behavior among women, particularly in terms of a male injecting his female partner, and also in terms of refusal to use condoms.

Respondents in our study demonstrated power emanating from marital dissolution or marital conflict. Knudson-Martin comments that among intimate partners, power “refers to the ability of one person to influence a relationship towards his own goals, interests and well-being” [36]. This is interesting for the fact that throughout their lives, WWUD in Malaysia are seen to be in a position of vulnerability and are often under the control of a man, making it significantly harder for harm reduction outreach services to reach them. Respondents made strong decisions, sometimes at the cost of being separated from their children, or being left without financial support. This places even stronger emphasis on harm reduction services already in contact with WWUD in unstable marriages to incorporate gender-sensitive counseling and other support services.

## Conclusion

The creation of environments conducive for intergenerational drug use accompanied by structural violence and loss of familial support networks complicate harm reduction services for WWUD. The most pertinent finding was the fact that familial instability was expressed as a gateway to drug use. In Malaysia, family is seen as the main support structure, and the absence of gender-sensitive and comprehensive welfare and health services for WWUD exacerbate the situation further. Interventions need to be more explicitly incorporate and address familial contributors to drug use, beginning with non-judgmental, evidence-based services for children who use drugs. Possible interventions that may be relevant are the following: ensuring that each harm reduction outreach service have trained female staff members, educating of mainstream healthcare providers on special needs of women who use drugs, parental rights services, and the incorporation of couples counseling, job placement, and skills training to help make WWUD become more independent, and thus helps address the power dynamics that increase HIV risk [37]. It is useful for Malaysian harm reduction practitioners to be mindful that the majority of our respondents used crystal methamphetamine, for which treatment is more difficult. A key limitation of the study is that our outreach workers who introduced respondents to the study could be known to social services for reasons other than drug use. Further qualitative and quantitative research on familial factors enabling drug use is necessary not only to inform modeling of gender sensitive services for Malaysian WWUD, but also to increase understanding on Southeast Asian WWUD and Muslim WWUD on the whole.

## Abbreviation

WWUD: women who use drugs
